# Inhibition of *Aspergillus Parasiticus* Growth and Aflatoxins Production by Natural Essential Oils and Phenolic Acids

**DOI:** 10.3390/toxins14060384

**Published:** 2022-05-31

**Authors:** Susana Lorán, Juan José Carramiñana, Teresa Juan, Agustín Ariño, Marta Herrera

**Affiliations:** 1 Instituto Agroalimentario de Aragón—IA2, Facultad de Veterinaria, Universidad de Zaragoza-CITA, 50013 Zaragoza, Spain; sloran@unizar.es (S.L.); carramin@unizar.es (J.J.C.); tjuan@cita-aragon.es (T.J.); herremar@unizar.es (M.H.); 2 Centro de Investigación y Tecnología Agroalimentaria de Aragón (CITA), 50059 Zaragoza, Spain

**Keywords:** antifungal, antiaflatoxigenic, essential oils, phenolic acids

## Abstract

Aflatoxins represent a significant risk to food safety, and strategies are being implemented to reduce their entry into the food chain. The aim of this study was to evaluate the in vitro effect of four essential oils (EOs) (lavandins *Grosso* and *Abrial*, *Origanum virens,* and *Rosmarinus officinalis*) and four natural phenolic acids (PAs) (caffeic, chlorogenic, ferulic, and *p*-coumaric) on the growth and aflatoxins (B1, B2, G1, and G2) production by *Aspergillus parasiticus*. Minimal inhibitory concentration (MIC) and minimal fungicide concentration (MFC) were determined by the broth macrodilution method. Additionally, the mycelia weight was determined at concentration levels lower than MIC. The antiaflatoxigenic activity was evaluated in the two concentrations of the EOs right before MIC and at concentrations below the MIC value for the PAs. To this end, in-house validated methodology based on high-performance liquid chromatography with post-column photochemical derivatization and fluorescence detection (HPLC-PHRED-FLD) was used. EOs of *O. virens* and lavandins (*Grosso* and *Abrial*) completely inhibited mold growth. In addition, a significant reduction in mycelial mass (*p* < 0.05) was observed for all EOs and PAs at different concentrations. In all cases except for lavandin *Abrial*, EO concentrations just before the MIC value strongly reduced (*p* < 0.05) aflatoxins synthesis. Aflatoxins production was completely inhibited by all PAs at a concentration of 20 mM; although at low concentrations, mycotoxin production was stimulated in some cases. The present study provides a scientific basis for further study of the inhibiting mechanisms.

## 1. Introduction

Mycotoxins are secondary metabolites produced by some filamentous fungi that occur naturally in food and feed. The presence of these compounds in the food chain is of great concern, due to their ability to induce acute and chronic toxic effects on animal and human health. The severity of mycotoxicoses depends on the toxicity of the mycotoxin, the degree of exposure, the age and nutritional status of the individual, as well as the possible synergistic effects of other chemical substances to which they may be exposed [1].

*Aspergillus parasiticus* is responsible for the production of aflatoxins B1, B2, G1, and G2. These are extremely toxic mycotoxins classified by the International Agency for Research on Cancer (IARC) as Group 1 carcinogens to humans [2]. They are produced pre- and post-harvest in certain conditions of temperature, water activity, and nutrient availability, and so, they are frequently found in many foodstuffs, such as cereals, nuts, cocoa, and oilseeds, among others.

Aflatoxin contamination of agricultural commodities has important economic implications since it can produce serious yield losses and cause both acute and chronic toxicity in animals and humans [3,4]. In addition, several reports seem to indicate that its incidence is increasing due to the effects of climate change [5]. For these reasons, different strategies both at pre- and post-harvest stages must be applied to reduce the aflatoxin impact in the food and feed chain.

For many years, a variety of different chemical and synthetic compounds has been used as antifungal agents to inhibit the toxigenic fungi. However, the need for green chemistry and sustainability has led to a renewal of scientific interest in the use of alternative methods for pest and disease control that produce minimal damage to the environment and human health, including the use of natural antifungal agents.

Essential oils (EOs) are volatile mixtures of organic compounds, including terpenes and terpenoids and aliphatic- and phenol-derived aromatic components. They are obtained from plant material by physical means such as steam distillation, which is most commonly used for commercial EO production [6]. They have demonstrated antioxidant, antifungal, and antibacterial potentials and food-preservative properties as well as low toxicity. In this sense, some EOs are listed as generally recognized as safe (GRAS) by the United States Food and Drug Administration, including lavandins, *Origanum* spp., and *Rosmarinus officinalis* [7]. Furthermore, several EOs have been reported as inhibitors of fungal growth and mycotoxin production in *Aspergillus* species [8,9,10]. Císarová et al. [8] reported that 15 tested essential oils exhibited antifungal and antiaflatoxin activity against three strains of *A. flavus* and *A. parasiticus*, with *A. flavus* showing the highest susceptibility. García-Díaz et al. [9] assayed the effectiveness of *Satureja montana* and *Origanum virens* essential oils to control *A. flavus* growth and toxin production. The growth of *A. flavus* was delayed by both essential oil treatments, but only *S. montana* essential oil was able to significantly affect aflatoxin production. The efficacy of eleven essential oils (EOs) against *A. flavus* was investigated in maize grains under vapor conditions [10]. The highest antifungal activity was exhibited by cinnamon, oregano, lemongrass, and their composite mixtures. The tested substances not only inhibited fungal growth and decreased aflatoxin production but also affected the colonization of *A. flavus* on maize grains.

In the same way, the vegetative growth and subsequent aflatoxins production by *Aspergillus* species were affected by phenolic acids (PAs), which are naturally present in vegetables such as grains and beans [11,12,13]. Bavaro et al. [11] reported that caffeic acid at 0.55 mM inhibited aflatoxin B1 production by 83% while increasing mycelial growth in *A. flavus*. Moon el al. [12] reported that the growth of *A. flavus* was completely inhibited with a concentration of ferulic acid equivalent to 50 mM, while at 5 mM and 0.5 mM, the inhibition was 67.2% and 14.2%, respectively. In another study with *A. westerdijkiae*, ochratoxin A (OTA) production was reduced by 35% by ferulic acid, while the fungal growth was unaffected [13]. Ahmed et al. [14] recently reviewed naturally occurring phenolic compounds as promising bioagents to inhibit fungal growth and/or to limit mycotoxin yields, including practical application of pathogen fungi control, especially for *Fusarium*. The most abundant PAs in cereal grains are hydroxycinnamic derivatives such as caffeic, chlorogenic, ferulic, and *p*-coumaric acids [15].

Hence, to evaluate the antifungal activity of natural compounds, it is important to determine their potential on the inhibition of fungal growth and toxin production before an extensive fieldwork is initiated. Thus, the aim of this work was to contribute to the knowledge of the activity of natural agents by evaluating the in vitro effects of four EOs (lavandin *Grosso*, lavandin *Abrial*, *Origanum virens,* and *R. officinalis*) and four PAs (caffeic, chlorogenic, ferulic, and *p*-coumaric acids) on the mycelial growth and aflatoxins production of the mycotoxigenic fungi *Aspergillus parasiticus*.

## 2. Materials and Methods

### 2.1. Fungal Strain and Inoculum Preparation

The aflatoxin-producing strain used in this study (*Aspergillus parasiticus* CECT 2682 able to synthetize the four major aflatoxins B1, B2, G1, and G2) was obtained from the Spanish Collection of Type Cultures (CECT, Valencia, Spain) and stored at –80 °C in cryovials. The mold was cultured in Sabouread broth (Oxoid, Hampshire, United Kingdom) and incubated for 4 days at 25 °C. After incubation, the revivified strain was grown on Potato Dextrose Agar (PDA) (Merck, Darmstadt, Germany) at 25 °C for 7 days. Spores were harvested by adding 10 mL of sterile distilled water containing 0.05% Tween 80 (Merck, Darmstadt, Germany) and scraping the surface of the culture grown on PDA slant tubes after 7 days of incubation at 25 °C. The spore suspension obtained was filtered through sterile gauze and then collected in sterile tubes. The number of viable spores/mL was determined by counting with a Neubauer chamber (0.0025 mm^2^, depth 0.1 mm) (Laboroptik Ltd., Lancing, UK) and confirmed by plate count on PDA incubated at 25 °C for 3 days. Finally, the spore suspension was further adjusted when needed with sterile distilled water to give a final concentration of 10^6^ spores/mL.

### 2.2. Natural Compounds

The essential oils (EOs) evaluated in this work, (i) lavandin *Grosso* and lavandin *Abrial* extracted from two hybrids of *Lavandula angustifolia* × *Lavandula latifolia*, (ii) *Origanum virens*, and (iii) *Rosmarinus officinalis,* were obtained from plants collected from experimental fields of the Agri-Food Research and Technology Center of Aragon (CITA, Zaragoza, Spain). The plants were mechanically harvested at full flowering and then air-dried. The following air-dried plant material was used: flowers in spikes from lavandins, leaves and flowers from *O. virens*, and terminal shoots and leaves from *R. officinalis*. The EOs were obtained by hydrodistillation in a Clevenger-type apparatus for 2 h. At the end of the distillation period, the oils were decanted into sterilized brown glass vials and stored at 4 °C in the dark when not in use. The composition of the EOs was determined by a gas chromatography-flame ionization detection (GC-FID) method in a recognized private laboratory.

Caffeic, chlorogenic, ferulic, and *p*-coumaric acids were obtained from Sigma-Aldrich Chemie GmbH (Taufkirchen, Germany). These phenolic acids (PAs) were selected because of their natural abundance in cereal grains and other plant foods. Stock solutions of PAs were daily prepared at 400 mM in 50% ethanol (Scharlab, Barcelona, Spain).

### 2.3. Effect of Natural Compounds on A. parasiticus Growth

Minimal inhibitory concentration (MIC) and minimal fungicide concentration (MFC) were determined by the broth macrodilution method in 10 mL test tubes. To evaluate MIC, experiments were performed in yeast extract sucrose (YES) broth containing 2% yeast extract (Merck, Darmstadt, Germany) and 15% sucrose (Merck, Darmstadt, Germany), with final pH adjusted to 5.5 [16]. The EOs were added at different concentrations (0.2, 0.4, 0.6, 0.8, 1, 3, and 5 µL/mL) dissolved with 3% ethanol in the YES broth. With regards to PAs, appropriate amounts of the stock solutions were added to have the working concentrations of 1, 5, 10, and 20 mM in the YES broth. Control treatments were prepared in the same manner with the equivalent amount of ethanol but without adding the tested compounds. All the tubes were inoculated with 100 µL of a 10^6^ spores/mL suspension, thoroughly mixed, and incubated at 25 °C for 10 days. MIC was determined as the lowest concentration of the added compounds, which was able to completely inhibit the visible growth of toxigenic strain of *A. parasiticus*. To evaluate MFC, 100 μL of the medium of each case in which fungal growth was not observed was subcultured on PDA plates and incubated for 7 days at 25 °C. The lowest concentration at which no growth occurred on the plates was taken as the MFC.

Additionally, the effect on the mycelial growth of *A. parasiticus* was evaluated by comparison of the weight of the mycelia obtained from those tubes with compounds under study at concentrations lower than the MIC values with that of the corresponding controls. In so doing, culture tubes were first subjected to a brief heat treatment (121 °C for 30 s) to inactivate spores and vegetative mycelia. Then, mycelia were separated from the broth by filtering through Whatman No. 4 filter paper (Symta, Madrid, Spain) and washed three times with 10 mL of distilled water. The filtrate was collected in test tubes for the subsequent analysis of aflatoxins B1 (AFB1), B2 (AFB2), G1 (AFG1), and G2 (AFG2). Finally, the mold mats were dried at 130 °C for 2 h, placed in a desiccator for 30 min, and the dry mycelia weighed [17].

### 2.4. Chemical and Reagents

The stock solution of aflatoxins was purchased from Sigma-Aldrich (St Louis, MO, USA) and consisted of a mix with 1 µg AFB1, 0.3 µg AFB2, 1 µg AFG1, and 0.3 µg AFG2 in methanol. Calibration curves for aflatoxins B1, B2, G1, and G2 were prepared at concentrations of 0.5, 1, 2.5, 5, and 10 ng/mL for AFB1 and AFG1 and 0.25, 0.5, 0.7, 1.5, and 3 ng/mL for AFB2 and AFG2. An intermediate solution for aflatoxins was made by 100-fold dilution of the original mix; then, working calibration solutions were prepared with mobile phase, stored at 4 °C, and renewed every week. Stock and intermediate standard solutions were stored at –20 °C. The immunoaffinity column (IAC) AflaTest WB SR was acquired from VICAM (Watertown, MA, USA). Deionized water was obtained from a Millipore milli-Q water purification system (Mildford, MA, USA). HPLC-grade acetonitrile and methanol were supplied from Scharlau (Scharlab, Barcelona, Spain), and sodium chloride was purchased from Panreac (Barcelona, Spain). 

As a safety note, all used laboratory glassware were treated with an aqueous solution of sodium hypochlorite (5%) before discarding to minimize health risks due to mycotoxin contamination [18].

### 2.5. HPLC Equipment and Chromatographic Conditions

The system consisted of an Agilent 1100 Series high-performance liquid chromatograph coupled to a micro vacuum degasser and a fluorescence detector (HPLC-FLD) (Agilent Technologies, Barcelona, Spain) with excitation and emission wavelength of 365 and 435 nm, respectively. Separation was carried out on a column Ace 5 C18, 250 × 4.6 mm, 5 µm particle size (Análisis Vínicos, Ciudad-Real, Spain). A manual injector system equipped with a 100 µL injector loop and a 250 µL syringe was used. The isocratic mobile phase for aflatoxins was methanol/acetonitrile/water (40:10:50, *v/v/v*), pumped with a flow rate of 1.0 mL/min. The retention times (min) for the analyzed aflatoxins were 9.95 for AFG2, 11.00 for AFG1, 13.51 for AFB2, and 15.09 for AFB1 (Supplementary Material Figure S1). The fluorescence intensity of AFB1 and AFG1 was improved with postcolumn photochemical derivatization using a photochemical reactor for enhanced detection (PHRED) (LCTech UVE, Dorfen, Germany) set at 254 nm. The identification and quantification of aflatoxins in the culture broth samples were performed using the software package OpenLAB CDC 2013 (Agilent Technologies, Barcelona, Spain).

### 2.6. Determination of Effect of Natural Compounds on Aflatoxins Production by A. parasiticus

Identification and quantification of aflatoxins B1, B2, G1, and G2 from the culture medium collected in the previous stage, was carried out by HPLC-PHRED-FLD. Previously, mycotoxins were extracted from the culture broth and the extract diluted and purified with immunoaffinity columns. The antiaflatoxigenic activity was evaluated in samples coming from test tubes with PAs in which visible growth of the mold were observed as well as in the two concentrations of the EOs assayed right before the corresponding MIC value. Briefly, 4 mL of homogenized culture medium added with 0.4 g of sodium chloride were extracted with 11 mL of methanol/water (80:20, *v*/*v*) using an T25 Ultra-Turrax—IKA homogenizer (Wilmington, NC, USA) for 1 min and filtered with Whatman No. 4 filter paper. A 10 mL aliquot portion was collected, diluted up to 40 mL with milli-Q water, and filtered with a glass-fiber filter paper (Symta, Madrid, Spain). Then, 10 mL of the diluted filtrate was passed through immunoaffinity cleanup column at a flow rate of 1–2 drops per second for purification purposes. The column was then rinsed with milli-Q water and dried before the elution of the aflatoxins with 1 mL of methanol. Later, 1 mL of milli-Q water was added to the eluate before filtering with 0.45 µm filter (Análisis Vínicos, Ciudad-Real, Spain) and injecting 100 µL into the HPLC- PHRED-FLD system.

The analytical procedure was validated in-house, according to Commission Regulation (EC) No. 401/2006 [19], in terms of linearity, sensitivity (limits of detection and quantification), precision (repeatability and reproducibility), and percentage of recovery. Linearity was assessed by constructing five-point calibration curves over the calibration range of 0.5 to 10 ng/mL for AFB1 and G1 and 0.25 to 3 ng/mL for AFB2 and G2. Linear regression lines were plotted using the peak area versus analyte concentrations and linearity was described by linear regression analysis and was expressed as coefficient of determination (R^2^) above 99%. The limits of detection (LD) and quantification (LQ) were determined for a signal/noise ratio of 3 and 10, respectively, using blank samples of sterile culture broth. These blank samples were spiked at different aflatoxin levels for recovery assays: 10 ng/mL (aflatoxins B1 and G1) and 3 ng/mL (aflatoxins B2 and G2) (Supplementary Material Figure S2). The precision was evaluated in terms of relative standard deviation (RSD%) from independent replicate analysis both intra-day and inter-day.

According to the results of the validation process, the analytical methodology provided good recoveries in the range of 78.2% (AFG1) to 94.6% (AFB2). In addition, precision in terms of repeatability (RSDr%) and reproducibility (RSD_R_%) presented values less than 11% and 14%, respectively, for all aflatoxins, in accordance with the performance criteria considered by Commission Regulation (EC) No. 401/2016 [19]. Sensitivity obtained was considered adequate for the purpose of the research, with limit of detection (LD) varying between 0.14 ng/mL (AFG2) and 0.36 ng/mL (AFG1) and limit of quantitation (LQ) ranging from 0.42 to 1.08 ng/mL for aflatoxins G2 and G1 as well. Reagents and blank samples that were analyzed at the beginning of the assay showed no background interferences with the analytical procedure. Additionally, for analytical quality control, our laboratory participated in worldwide interlaboratory comparison rounds organized by Romer Labs during 2017 (Ref. CSSMY013-M17411A) and 2019 (CSSMY017-M19411AF), obtaining satisfactory results in terms of z-score.

### 2.7. Statistical Analysis

The evaluation of MIC and MFC was carried out in triplicate and the modal value selected [20]. Experiments on the effect of natural compounds on the mycelial growth and aflatoxins (B1, B2, G1, and G2) production by *A. parasiticus* were performed three times using freshly prepared samples. Mean and standard deviations were calculated from all the data obtained in the experiments performed. Data were statistically evaluated by *t*-test (*p* < 0.05) to determine whether the means of two groups are equal to each other (control against treatment with essential oils or phenolic acids). All the statistical analyses were performed using the IBM SPSS Statistics Base program, version 22 (Armonk, NY, USA).

## 3. Results

### 3.1. Effect of Natural Compounds on A. parasiticus Growth

Eight natural compounds, four essential oils (EOs), and four phenolic acids (PAs) were evaluated in vitro against a mycotoxigenic strain of *Aspergillus parasiticus* by the broth macrodilution method. Results showed variations in the antifungal properties of the assayed compounds depending on the concentration and type of substance.

EOs were tested for their ability to inhibit the growth of *A. parasiticus* at different concentrations, namely 0 (control), 0.2, 0.4, 0.6, 0.8, 1, 3, and 5 µL/mL in YES broth, and incubated for 10 days when the stationary phase of growth was reached. *Origanum virens* and lavandins (*Grosso* and *Abrial*) EOs were able to completely inhibit the mold growth. The most effective one was *O. virens* oil, with minimal inhibitory concentration (MIC) and minimal fungicide concentration (MFC) values of 0.6 µL/mL. EOs from lavandin *Grosso* and lavandin *Abrial* showed similar activity: the same MIC value of 3 µL/mL for both, while the MFC values were 3 µL/mL for *Grosso* and 5 µL/mL for *Abrial*, respectively. *Rosmarinus officinalis* oil, at all concentrations tested, was not able to completely inhibit the *A. parasiticus* growth (MIC and MFC > 5 µL/mL) (Table 1).

**Figure 1:**
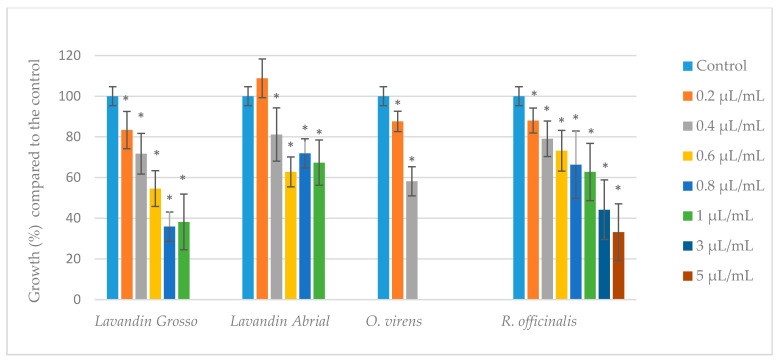
Effect of lavandin *Grosso*, lavandin *Abrial, O. virens,* and *R. officinalis* essential oils on mycelial growth of *A. parasiticus* cultured in yeast extract sucrose (YES) broth at 25 °C for 10 days. Values expressed as mean values ± standard deviation (SD) of three independent replications. The sign * indicates a significant difference with respect to controls according to *t*-test (*p* < 0.05).

As a reference, the mycelial dry weight for the untreated control was 0.367 g ± 0.018 at 10 days of incubation. For a better comparison, Figure 1 shows the variations expressed in percentage change of mycelial dry weight relative to the control (100%) with the different EOs. Concentrations lower than MIC values showed a variable effect on the mycelial growth of *A. parasiticus*, depending on the oil (Figure 1). Particularly, lavandin *Abrial* oil stimulated *A. parasiticus* mycelial growth at the lowest concentration assayed (0.2 µL/mL). However, it should be noted that a significant reduction (*p* < 0.05) of mycelial mass for lavandin *Grosso* oil from 0.2 to 1 µL/mL, lavandin *Abrial* oil between 0.4 and 1 µL/mL, *O. virens* oil at 0.2 and 0.4 µL/mL, and *R. officinalis* oil at 0.2 to 5 µL/mL was observed. For mycelial dry weight, the EOs interfered with the normal growth processes of *A. parasiticus*, and the inhibitory effect was enhanced when the concentration was increased. This might be because of the damage to the cell wall and cell membrane caused the blockage of the cell growth and the mycelia decline, which led to the weight of the mycelia being reduced significantly.

**Figure 2:**
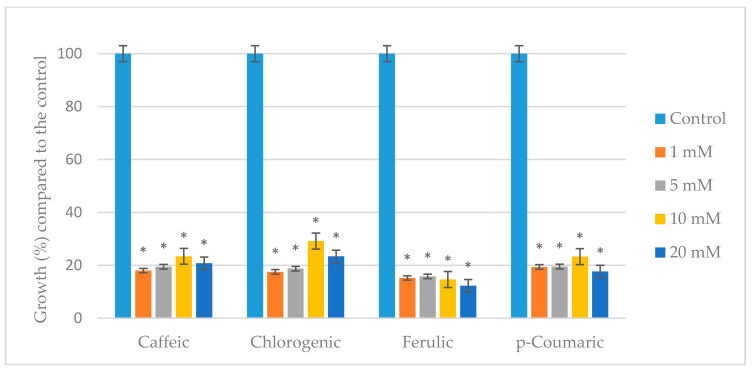
Effect of caffeic, chlorogenic, ferulic, and *p*-coumaric phenolic acids on mycelial growth of *A. parasiticus* cultured in YES broth at 25 °C for 10 days. Values expressed as mean values ± SD of three independent replications. The sign * indicates a significant difference with respect to controls according to *t*-test (*p* < 0.05).

PAs were also tested for their ability to inhibit the growth of *A. parasiticus* at different concentrations, namely 0 (control), 1, 5, 10, and 20 mM (roughly corresponding to from 0.2 to 4 mg/mL) in YES broth, incubated for 10 days when the stationary phase of growth was reached. In view of the results, EOs were more effective than PAs in controlling the growth of *A. parasiticus*. Nevertheless, none of the four PAs was able to completely inhibit the growth of the fungi, so the MIC and MFC values were set at more than 20 mM. However, at concentrations below the MIC, all PAs produced significant reductions in mycelial growth greater than 70% (*p* < 0.05), with ferulic acid being the most effective (Figure 2). For a reference here, the mycelial dry weight for the untreated control was 0.397 g ± 0.012 at 10 days of incubation. For comparability, Figure 2 shows the variations expressed in percentage change of mycelial dry weight relative to the control (100%) with the different PAs. The antifungal activity of the four PAs was very similar in all the concentrations under study, so it was not possible to establish a clear relationship between the percentage of inhibition of mycelial growth and the tested concentrations. What is more, in all cases but for ferulic acid, smaller reduction in mycelial growth was observed at the highest concentrations assayed (10 mM and 20 mM) when compared to the lowest ones evaluated (1 mM and 5 mM) although these differences were not very remarkable. For mycelial dry weight, the PAs negatively affected the normal growth processes of *A. parasiticus*. Phenolic acids can interact with the fungal membrane, which could in turn affect the fungal development leading to reduced mycelial dry weight.

### 3.2. Effect of Natural Compounds on Aflatoxins Production by A. parasiticus

Aflatoxins B1 (AFB1), B2 (AFB2), G1 (AFG1), and G2 (AFG2), present in the filtrate culture medium collected, were analyzed by in-house validated methodology based on high-performance liquid chromatography and post-column photochemical derivatization with fluorescence detection (HPLC-PHRED-FLD). For calculations, the concentrations of the four aflatoxins in the untreated controls were in the range of AFB1 0.52 µg/mL, AFB2 0.09 µg/mL, AFG1 0.94 µg/mL, and AFG2 0.26 µg/mL, with an average proportion of 28.7% AFB1, 5.0% AFB2, 51.9% AFG1, and 14.4% AFG2.

**Figure 3:**
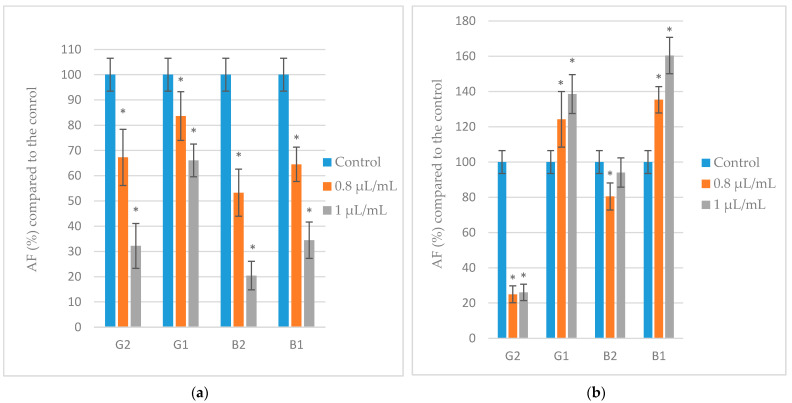

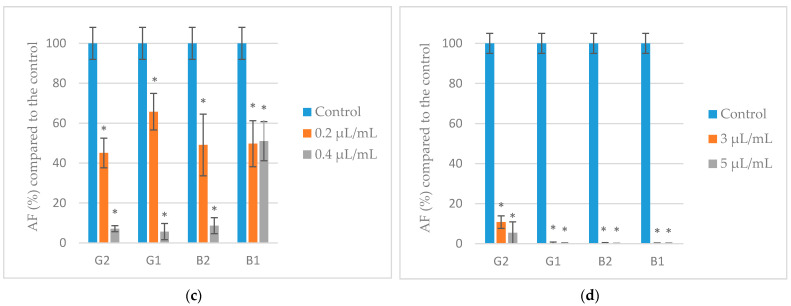
Effect of lavandin *Grosso* (**a**), lavandin *Abrial* (**b**), *O. virens* (**c**) and *R. officinalis* (**d**) essential oils on aflatoxins (AF) production by *A. parasiticus* cultured in YES broth at 25 °C for 10 days. Values expressed as mean values ± SD of three independent replications. The sign * indicates a significant difference with respect to controls according to *t*-test (*p* < 0.05).

Differences between treated and untreated control make it possible to estimate the percentage of mycotoxin yield inhibition. For a suitable comparison, Figure 3 shows the variations expressed in percentage change in the concentration of each aflatoxin relative to its respective control (100%) with the different EOs. When evaluating the effect of the two concentrations of essential oils (EOs) lower than the MIC values, a significant reduction was observed in the synthesis of aflatoxins (B1, B2, G1, and G2) compared to the controls (Figure 3).

Regarding the essential oil of *R. officinalis*, it is noteworthy that although it was not able to completely reduce the growth of *A. parasiticus*, it did almost completely inhibit the production of aflatoxins (*p* < 0.05), showing reductions with respect to the controls above 89% for AFG2 and above 99% for the other aflatoxins (B1, B2, and G1). Reduction of mycotoxin production was found to require lower concentrations of rosemary EO than growth inhibition. Perhaps it is another example that fungal growth and mycotoxin production are not affected in the same way by the different compounds contained in plant extracts.

When comparing to the controls, lavandin *Grosso* and *O. virens* EOs significantly prevented (*p* < 0.05) the synthesis of aflatoxins (B1, B2, G1, and G2) in a concentration-dependent manner. In turn, lavandin *Abrial* oil did not have a significant effect since it even stimulated the production of AFB1 and AFG1 at the two concentrations evaluated (0.8 µL/mL and 1 µL/mL). This oil was only able to significantly reduce (*p* < 0.05) the percentage of AFB2 (at 0.8 µL/mL) and AFG2 (at 0.8 µL/mL and 1 µL/mL).

**Figure 4:**
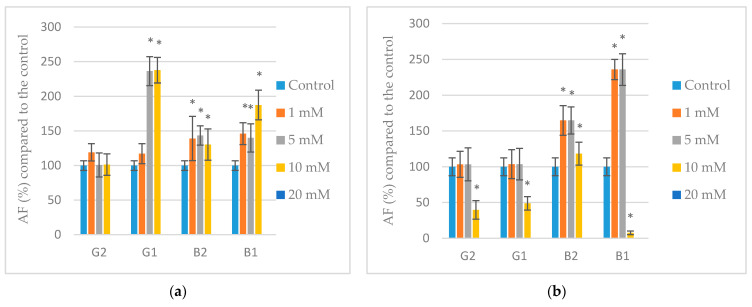

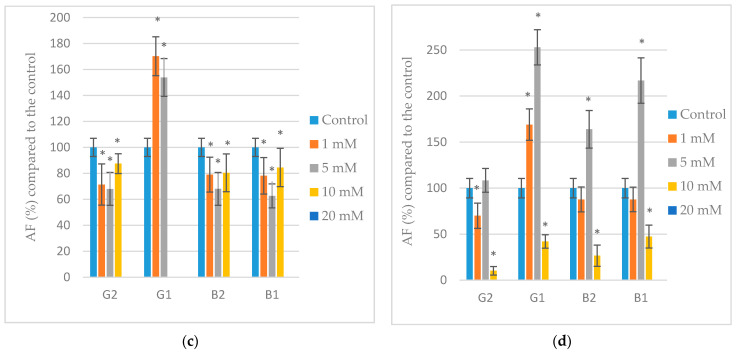
Effect of caffeic (**a**), chlorogenic (**b**), ferulic (**c**), and *p*-coumaric (**d**) acids on aflatoxins (AF) production by *A. parasiticus* cultured in YES broth at 25 °C for 10 days. Values expressed as mean values ± SD of three independent replications. The sign * indicates a significant difference with respect to controls according to *t*-test (*p* < 0.05).

The validated method was also used to determine the inhibition of aflatoxin synthesis by phenolic acids (PAs) at concentrations below the MIC value. Figure 4 shows the variations expressed in percentage change in the concentration of each aflatoxin relative to its respective control (100%) with the different PAs. The results showed a variable effect depending on the added concentrations of PAs. As shown in Figure 4, aflatoxins production by *A. parasiticus* was completely inhibited at the highest concentration tested (20 mM) by the four PAs assayed. Apart from this, the rest of concentrations evaluated hardly inhibited the aflatoxins production when comparing to the controls except in some cases (10 mM of chlorogenic, ferulic, or *p*-coumaric acids). Moreover, a significant increase in production (*p* < 0.05) of certain aflatoxins could be observed when 5 mM of caffeic (aflatoxins B1, B2, and G1), chlorogenic (aflatoxins B1 and B2), ferulic (aflatoxin G1), or *p*-coumaric (aflatoxins B1, B2, and G1) acids were added to the culture medium. A stimulating effect (*p* < 0.05) on aflatoxins production was also exhibited by PAs at 1 mM: caffeic (aflatoxins B1, B2, G1, and G2), chlorogenic (aflatoxins B1 and B2), ferulic (aflatoxin G1), or *p*-coumaric (aflatoxin G1).

### 3.3. Chemical Composition of Essential Oils

The main components and their relative proportions in EOs were determined by a GC-FID method performed in the external laboratory INSERCO Laboratorios (Zaragoza, Spain), which is accredited by the Spanish National Accreditation Body (ENAC) under the UNE-EN ISO-17025 standard to undertake the analysis. The laboratory uses an Agilent apparatus equipped with OpenLab CDS chromatography data system using EZChrom Elite 3.2.0 software. For the chemical composition of all tested EOs, the GC peaks were identified by comparing their retention times with those of reference standards available in the laboratory (from Merck Life Science S.L.U., Madrid, Spain). Component relative concentrations were obtained directly from GC peak areas compared with external calibration curves developed by injecting different amounts of authentic standards in the GC system (Table 2).

Lavandin *Grosso* oil was primarily composed of linalool (31.65%), linalyl acetate (24.98%), 1,8-cineole (8.69%), camphor (6.96%), and terpinen-4-ol (3.10%). In lavandin *Abrial* oil, linalool (31.04%), linalyl acetate (19.57%), 1,8-cineole (10.46%), camphor (8.86%), and (E)-β-ocimene (3.50%) were the major components identified. In *O. virens* oil, carvacrol (28.71%) was the most abundant compound, followed by *p*-cymene (9.55%), Ƴ-terpinene (5.22%), α-terpinene (3.00%), myrcene (2.05%), and thymol (1.78%). The primary components identified in *R. officinalis* oil were α-pinene (21.01%), 1,8-cineole (20.06%), camphor (10.91%), camphene (7.07%), myrcene (5.33%), bornil acetate (4.02%), and p-cymene (3.77%).

## 4. Discussion

Many preventive strategies have been developed to reduce mycotoxin occurrence in food commodities. These strategies range from good agricultural practices to the use of biocontrol agents or natural compounds able to avoid toxin production [21]. The search for new natural products to control either spoilage or toxigenic molds is a promising area of research. Therefore, studies contributing to the knowledge of the effect of these compounds in different fungi that can threat public health are still required. The results reported in this work demonstrate the potential of some essential oils (EOs) and phenolic compounds to inhibit *A. parasiticus* growth and its aflatoxins production.

EOs are volatile substances naturally produced by plants that may provide an alternative method to protect food and feed from fungal contamination. Consistent with our results, other studies have also shown that EOs have the capability on inhibiting both fungal growth and mycotoxin production in *Aspergillus* species [22,23,24,25,26]. In this experiment, EOs were the most effective substances to control the growth and aflatoxins production of *A. parasiticus* since they were able to completely inhibit the mold, with the exception of *Rosmarinus officinalis* (with MIC and MFC > 5 μL/mL). Similar results have been previously found in the study of antifungal and antiaflatoxigenic action of the rosemary essential oil (EO), ranging from 1.25 to > 5 μL/mL [27]. Consequently, some authors reported that rosemary EO was more effective against other *Aspergillus* species such as *A. niger*, while it failed to control *A. flavus*, *Penicillium minioluteum*, or *Penicillium oxalicum* [28].

On the other hand, a significant reduction of mycelial mass for all EOs at concentrations lower than MIC values was observed. Furthermore, all EOs but lavandin *Abrial* oil were able to strongly reduce the synthesis of aflatoxins in the two concentrations right before MIC values. In the present study, lavandin *Abrial* was the only essential oil (EO) that stimulated, at low concentrations, fungal growth (at 0.2 µL/mL) and mycotoxin production (AFB1 and AFG1 at 0.8 and 1 µL/mL) of *A. parasiticus*. In this sense, other authors suggested that low fungicide doses create some stress conditions that may be responsible for the production of more secondary metabolites as a defense mechanism by the fungus [29,30,31,32]. Therefore, controlling the dose of fungicides is crucial since suboptimal concentrations could lead to stimulation of both growth and toxin accumulation [30].

Fungal growth and mycotoxin production may not be affected in the same way by the different compounds contained in essential oils. It was particularly remarkable that while essential rosemary oil (*R. officinalis*) did not inhibit fungal growth at MIC/MFC < 5 µL/mL, it showed the strongest inhibition of aflatoxin production. Essential oils are a valuable source of bioactive natural products in the fight against toxigenic fungi, as they can affect fungal development and/or the synthesis of mycotoxins. However, the mechanisms involved in these two phenomena are not yet well-known. For instance, Prakash et al. [27] identified plasma membrane as the target action site for antifungal activity of rosemary EO, which alters membrane permeability and functioning, leading to cell death. Safari et al. [33] reported that the inhibitory mechanisms of certain plant extracts were related to their effect on gene expression responsible for aflatoxin B1 biosynthesis. Their results showed that *Heracleum persicum*, *Peganum harmala,* and *Trachyspermum ammi* completely stopped the production of aflatoxin B1, without inducing significant changes in *A. flavus* growth, which suggests that the mechanisms involved in the regulation of fungal growth and mycotoxin synthesis could be different.

Chemical composition of plant EOs differ among species; it is affected by factors including the geographical location, environment, the stage of maturity, and method of extraction [34]. The chemical composition of lavandins, oregano, and rosemary have been reported earlier [35,36,37,38]. Although the proportion of some component differed from previous studies, the main chemical composition was the same as found in our analyses. In general, the potential of plant essential oils to inhibit fungal growth depends on their chemical composition, both qualitative and quantitative. Essential oils are usually rich in various compounds, comprising 20 to 60 active substances, and, in many cases, can be characterized by up to three major components at a relatively high concentration compared to other compounds present in trace amounts. The major components found in EO are often responsible for their biological properties [39]. In our study, *Origanum virens* EO was the most effective for inhibiting growth of *A. parasiticus* (MIC and MFC values of 0.6 µL/mL). Accordingly, Viuda-Martos et al. [40] also showed that the antifungal activity of *O. virens* is much stronger than other EOs. Antifungal and antimycotoxigenic effect of oregano EO used in this study might be due to the reaction of its oxygenated monoterpenes (such as carvacrol and thymol) with reactive groups of fungal enzymes [41], possibly through reactions with sulfhydryl groups or through non-specific interactions with proteins. Their possible consequence is damage to membrane integrity, which could affect pH homeostasis and equilibrium of inorganic ions. However, it is difficult to establish a relationship between EO composition and biological activity because of the synergistic actions between various components. It is indeed well-known that EOs have a higher antifungal activity than does a mixture of their major components, which suggests that the minor components are critical to the activity and may contribute to a synergistic effect [42].

Phenolic acids (PAs) are naturally present in the outer layers of grains, and they have been reported as inhibitors of fungal growth and mycotoxin production in *Aspergillus* species [43]. However, large differences in the response of toxic molds to phenolic compounds are observed in the reviewed literature according to the phenolic compound itself, the mycotoxin studied, and the producing fungal strain [44]. In addition, it has been suggested that they may also be due to differences in experimental conditions, among which water stress seems to be especially important [45]. Among hydroxycinnamic acid derivatives, chlorogenic acid and its hydrolyzed product, caffeic acid, have shown a strong antimycotoxin effect at low concentration against a range of mycotoxins, including aflatoxin B1 [11]. Transcriptomic approaches indicated that caffeic acid negatively impacted the expression of aflatoxin biosynthesis key genes of *A. flavus*. Ferulic acid, a methylated form of caffeic acid, could also affect the growth of toxigenic *A. flavus* [12]. Ahmed et al. [14] recently reviewed the mechanisms involved in antifungal and antimycotoxin action of phenolic compounds. They include the following: fungal membrane modifications affecting permeability and functioning, reduction in oxidative stress and inhibition of oxidases, as well as downregulation of the expression of key genes involved in mycotoxin production.

Regarding the present work, *A. parasiticus* showed susceptibility to all PAs when exposed for 10 days although none of them was able to completely inhibit mold growth. The phenolic acids can disrupt the permeability of the fungal membrane by interacting with lipid bilayers, resulting in altered homeostasis through leakage of intracellular constituents and growth impairment [46]. However, despite the great inhibition of mycelial growth achieved at any of the concentrations below the MIC values (more than 70%), only the highest concentration of 20 mM significantly reduced aflatoxin production, while lower concentrations showed a variable effect, even stimulating the biosynthesis of aflatoxins. In summary, reduction of fungal growth was found to require lower concentrations of PAs than mycotoxin production. Similar to our results, Nesci and Etcheverry [45] found that trans-cinnamic and ferulic acids were effective in preventing *A. flavus* and *A. parasiticus* growth and aflatoxin B1 (AFB1) formation when applied at different concentrations on 3% maize meal extract agar. In agreement with our results, they also observed that under some conditions, low concentrations of PAs stimulated growth and toxin production. In our case, no growth stimulation was observed though aflatoxins biosynthesis was encouraged in some cases at low concentrations.

These results suggest that there is not such a direct relationship between PAs and growth inhibition or mycotoxin production. However, other authors have supported a different idea [47,48] suggesting that, in the case of *Fusarium* species, the inhibition of mycotoxin production by natural PAs is related to growth inhibition. At this point, it is important to consider that the exact mechanism of action by which PAs are able to repress mycotoxin biosynthesis has not been completely elucidated yet [49]. Compounds that inhibit mycotoxin production can act by altering the environmental and physiological modulators of mycotoxin biosynthesis or by altering the signal transduction pathways upstream of the biosynthetic pathway [50]. A few studies already demonstrated that some of these natural products can inhibit AFB1 production by a transcriptional down-regulation of the genes involved in AFB1 synthesis [51,52].

The present results open new perspectives to the targeted search of naturally occurring compounds that may find practical application in the eco-friendly control of toxigenic fungi and mycotoxins to ensure a safe food and feed supply.

## 5. Conclusions

The results of the present study confirm the inhibitory effect of various essential oils (EOs) and phenolic acids (PAs) at appropriate concentrations on the growth and aflatoxins production of *A. parasiticus*. EOs were the most effective compounds since, with the exception of *Rosmarinus officinalis* oil, they were able to completely inhibit mold growth. The most effective one was *Origanum virens* oil, with MIC and MFC values of 0.6 µL/mL. In addition, a significant reduction of mycelial mass for all EOs was observed at concentrations lower than MIC values. Furthermore, all EOs but lavandin *Abrial* were able to strongly reduce the synthesis of aflatoxins in the two concentrations right before MIC values.

Regarding PAs, none was able to completely inhibit the mold growth. However, significant inhibition percentages of mycelial growth, higher than 70%, were obtained with the four PAs assayed at concentrations lower than MIC values. Aflatoxins production was entirely inhibited at concentration of 20 mM by all PAs although, in some cases, at low concentrations, they stimulated mycotoxin production. Therefore, controlling the dose of natural fungicides is crucial since suboptimal concentrations could lead to stimulation of both growth and toxin accumulation. In any case, EOs and PAs may provide an alternative strategy to the use of synthetic chemical fungicides to ensure food safety, which deserves additional studies to assess more details regarding their practical applications.

## Figures and Tables

**Table 1:** Antifungal activity (MIC and MFC values) of lavandin *Grosso*, lavandin *Abrial, O. virens,* and *R. officinalis* essential oils against *A. parasiticus* cultured in YES broth at 25 °C for 10 days. The evaluation of MIC and MFC was carried out in triplicate and the modal value selected.

Essential Oil	MIC (µL/mL)	MFC (µL/mL)
Lavandin *Grosso*	3	3
Lavandin *Abrial*	3	5
*Origanum virens*	0.6	0.6
*Rosmarinus officinalis*	>5	>5

**Table 2:** Main components of lavandin *Grosso*, lavandin *Abrial*, *Origanum virens,* and *Rosmarinus officinalis* essential oils by gas chromatography, expressed in percentage (%).

Compound	Lavandin *Grosso*	Lavandin *Abrial*	Compound	*Origanum* *Virens*	*Rosmarinus* *Officinalis*
Linalool	31.65	31.04	Carvacrol	28.71	nd
Linalyl acetate	24.98	19.57	P-cymene	9.55	3.77
1,8-cineole	8.69	10.46	γ-terpinene	5.22	nd
Camphor	6.96	8.86	α-terpinene	3.00	nd
Terpinen-4-ol	3.10	0.85	Myrcene	2.05	5.33
Borneol	2.44	2.60	Thymol	1.78	nd
Lavandulyl acetate	2.37	1.90	β-caryophillene	1.44	nd
Cis-β-farnesene	1.18	0.60	α-thujene	1.19	nd
Limonene	1.13	1.22	Terpinen-4-ol	0.60	0.75
Cis-β-ocimene	0.56	2.01	α-pinene	0.49	21.01
(e)-β-ocimene	0.20	3.50	Linalool	0.30	0.88
Lavandulol	nd ^1^	0.50	1,8-cineole	nd	20.06
			Camphor	nd	10.91
			Camphene	nd	7.07
			Bornil acetate	nd	4.02
			Borneol	nd	1.85
			β-pinene	nd	1.26
			Verbenone	nd	1.19
			α-terpineol	nd	1.07

^1^ not detected.

## Data Availability

The data presented in this study are available in article.

## Supplementary Materials

The following supporting information can be downloaded at: www.mdpi.com/article/10.3390/toxins14060384/s1, Figure S1: Chromatogram of a standard solution of aflatoxins at 5 ng/mL (aflatoxins B1 and G1) and 1.5 ng/mL (aflatoxins B2 and G2); Figure S2: Chromatogram of a blank (YES broth) spiked with 10 ng/mL (aflatoxins B1 and G1) and 3 ng/mL (aflatoxins B2 and G2).
