# Supplementary material for: Inhibition of Aspergillus Parasiticus Growth and Aflatoxins Production by Natural Essential Oils and Phenolic Acids
Source: Toxins (Basel). 2022 May 31;14(6):384. doi: 10.3390/toxins14060384 (PMC9227641; doi:10.3390/toxins14060384)
Supplement: Supplementary file 1 [file toxins-14-00384-s001.zip › toxins-1721388-supplementary.pdf]

Supplementary Materials: Inhibition of *Aspergillus Parasiticus* Growth and Aflatoxins Production by Natural Essential Oils and Phenolic Acids

Susana Lorán, Juan José Carramiñana, Teresa Juan, Agustín Ariño and Marta Herrera

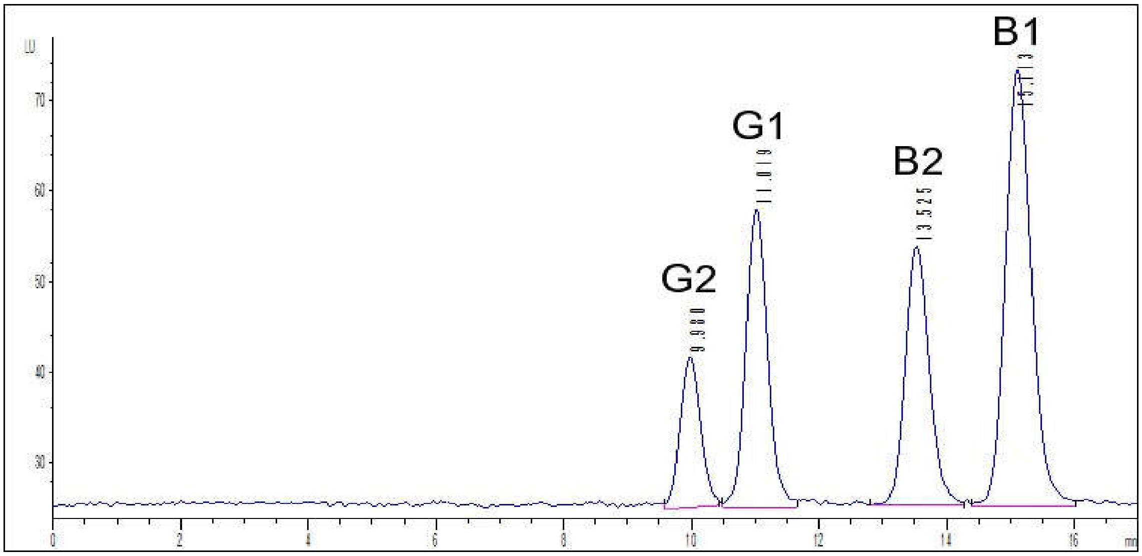

Figure S1. Chromatogram of a standard solution of aflatoxins at 5 ng/mL (aflatoxins B1 and G1) and 1.5 ng/mL (aflatoxins B2 and G2). Amount injected AFB1 0.50 ng, AFB2 0.15 ng, AFG1 0.50 ng, AFG2 0.15 ng.

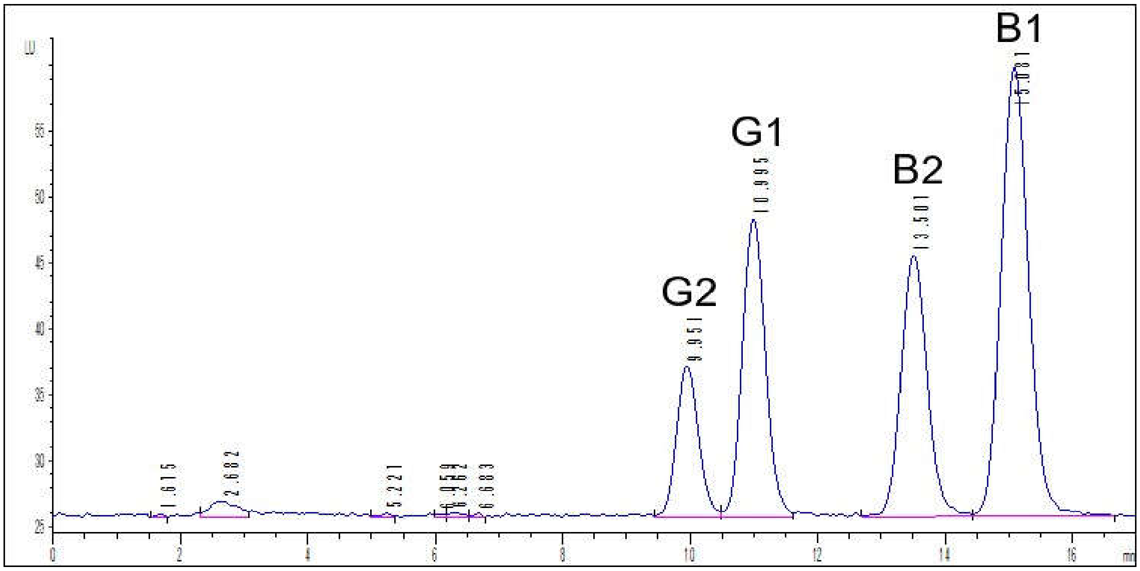

Figure S2. Chromatogram of a blank (YES broth) spiked with 10 ng/mL (aflatoxins B1 and G1) and 3 ng/mL (aflatoxins B2 and G2). Amount injected AFB1 0.33 ng, AFB2 0.10 ng, AFG1 0.33 ng, AFG2 0.10 ng.
